# BrainYOLO-MCA: An Improved YOLOv11 with Multi-Scale Channel Attention for Brain Tumor Detection

**DOI:** 10.2174/0115734056507718260630052655

**Published:** 2026-07-07

**Authors:** Ran Xie, Jingang Ma, Yang Li

**Affiliations:** 1 School of Medical Information Engineering, Shandong University of Traditional Chinese Medicine, Jinan, China

**Keywords:** Brain tumor detection, YOLOv11, Multi-scale channel attention, MRI, Deep learning, Object detection, BrainYOLO-MCA

## Abstract

**Introduction:**

Brain tumor detection in magnetic resonance imaging is crucial for early diagnosis and clinical treatment planning; however, it remains a challenging task due to the difficulty in detecting tumors with blurred boundaries, high visual similarity to normal brain tissue, and tiny early-stage lesions. To address these limitations, this study proposes a novel brain tumor detection framework named BrainYOLO-MCA.

**Methods:**

BrainYOLO-MCA is an improved YOLOv11 architecture with a core design of a novel Multi-scale Channel Attention (MCA) mechanism to enhance multi-scale feature representation and fuse critical texture and background information, thereby improving the recognition of indistinct tumor boundaries. On this basis, three targeted optimizations were integrated: a dynamic upsampling strategy (DySample) to preserve the spatial details of tiny lesions, an additional small-object detection head to enhance sensitivity to early-stage small tumors, and a sparse self-attention mechanism to strengthen global contextual feature representation. The proposed model is comprehensively validated on three public brain tumor datasets (Br35H, MBrT, Figshare).

**Results:**

Experimental results show that the study’s method achieves 98.6% precision and 97.5% mAP50 on Br35H, 95.3% precision and 97.5% mAP50 on MBrT, and 96.1% precision and 96.3% mAP50 on Figshare, outperforming mainstream YOLO-based models including the baseline YOLOv11, while maintaining a lightweight architecture with only 3.1M parameters.

**Discussion:**

These results demonstrate that the proposed model improves the detection of tumors with blurred boundaries and tiny lesions, while achieving consistent performance across multiple datasets. The Multi-scale Channel Attention and feature fusion strategies enhance feature representation and improve robustness and detection accuracy.

**Conclusion:**

The proposed BrainYOLO-MCA enables more accurate and reliable brain tumor detection, which can support clinical decision-making and facilitate early diagnosis of brain tumors.

## 1 INTRODUCTION

Brain tumors are space-occupying intracranial lesions caused by uncontrolled proliferation of abnormal intracranial tissue cells, which can compress vital brain structures, impair central nervous system function, and induce severe, even life-threatening neurological complications [1]. Based on pathological characteristics, brain tumors are broadly categorized into benign, precancerous, and malignant subtypes [2, 3]. Benign lesions typically have well-defined margins and are treatable *via* surgical resection, while malignant tumors are highly invasive, prone to metastasis, and associated with extremely poor clinical prognosis [2, 4]. Timely and accurate early detection is the cornerstone of effective clinical intervention, which can significantly improve treatment outcomes and long-term survival of patients [3, 4].

Medical imaging is the primary non-invasive tool for clinical brain tumor detection [5, 6], among which Magnetic Resonance Imaging (MRI) is widely recognized as the gold standard for brain tumor imaging, with superior soft tissue contrast, multi-planar imaging capability, and no ionizing radiation [7, 8]. However, accurate MRI-based brain tumor detection remains clinically challenging: lesions with blurred boundaries, high similarity to normal brain parenchyma, and tiny early-stage tumors are prone to missed detection and false positives, even for experienced radiologists [9]. Early computer-aided detection systems were mainly based on conventional machine learning methods, most notably Support Vector Machines (SVM) [10] and random forests [11], whose performance is severely limited by dependence on hand-crafted features and poor generalization for complex tumor morphology [11]. With the advancement of deep learning, object detection algorithms have gradually become the core of intelligent diagnostic systems, which can be broadly categorized into two primary architectural paradigms [12-15]. Two-stage detectors, represented by Faster R-CNN [12], achieve high detection accuracy but incur substantial computational overhead, making them unsuitable for real-time clinical applications [13]. One-stage detectors, represented by SSD [14] and the YOLO series [15], achieve a better trade-off between detection precision and inference speed, making them well-suited for clinical scenarios.

In recent years, the YOLO family has been extensively applied to medical image analysis, including brain tumor MRI detection [16]. For brain tumor detection specifically, an Enhanced Spatial Attention (ESA) layer has been integrated into YOLOv5m to improve brain tumor subtype differentiation [17]; a YOLOv8-based detection algorithm with a large-kernel re-parameterized convolution network has been proposed [18]; YOLO-NeuroBoost with dynamic convolution kernels has been developed for high-accuracy brain tumor detection [19]; and RCS-YOLO, a modified YOLO architecture optimized for brain tumor detection, has been designed [20]. Meanwhile, feature pyramid and attention mechanisms have been widely adopted to enhance detection performance in medical image analysis [15]. However, existing YOLO-based methods still have three critical limitations for clinical brain tumor detection: first, most attention modules neglect non-semantic texture features critical for identifying boundary-blurred lesions [21]; second, tiny early-stage tumors suffer from severe feature loss during downsampling and traditional upsampling operations [15]; third, full self-attention mechanisms introduce redundant computation and limit the model’s deployment on clinical edge devices [22].

Based on the limitations of existing methods, this study proposes the following hypotheses: The multi-scale channel attention mechanism can improve the recognition ability of tumors with blurred boundaries; Dynamic upsampling and high-resolution detection heads can enhance the detection sensitivity of small lesions; Sparse self-attention can strengthen global contextual features without significantly increasing computational cost. The core design of BrainYOLO-MCA is the novel MCA module, which combines average pooling and max pooling to preserve background context and texture details, and extracts multi-scale features *via* parallel multi-scale convolutions, effectively enhancing the recognition of boundary-blurred tumors. On this basis, three targeted optimizations are integrated into the framework: a DySample dynamic upsampling strategy to preserve spatial details of tiny lesions, an additional dedicated small-object detection head to improve sensitivity to early-stage small tumors, and a sparse self-attention mechanism to strengthen global contextual feature representation with reduced computational complexity. We comprehensively validate the proposed model on three public brain tumor MRI datasets: Br35H, MBrT, and Figshare. Experimental results show that our model outperforms mainstream YOLO-based approaches, including the original YOLOv11 baseline, across all core evaluation metrics, while maintaining a lightweight architecture with only 3.1M parameters. The improved YOLOv11 provides an efficient and reliable computer-aided diagnosis tool for clinical brain tumor detection, with the potential to reduce missed diagnoses and support clinical decision-making for early tumor intervention.

## 2 MATERIALS AND METHODS

### 2.1 Dataset

All data used in this study were obtained from three publicly available annotated brain MRI datasets: the Br35H dataset [23], the MBrT dataset [24], and the Figshare dataset [25]. All datasets had been de-identified and contained no personally identifiable information. In addition, a unified standardization preprocessing procedure was applied to all data, including resizing all images to 640 × 640 pixels and normalizing pixel values to the range from 0 to 1. For all datasets, the training, validation, and test subsets were randomly divided at an 8:1:1 ratio. These datasets include both binary and multi-class brain tumor MRI images with varying tumor categories and imaging characteristics, providing diverse evaluation scenarios for assessing model robustness and generalization. Detailed dataset statistics and class distributions are summarized in Table **1**, while representative samples are shown in Fig. (**1**). During the training phase, Mosaic augmentation, random horizontal flipping, random rotation within ±15°, and color augmentation were used. During the validation and testing phases, only image size normalization to 640×640 and pixel value standardization were performed.

### 2.2 Methods

#### 2.2.1 Overall Architecture

YOLOv11, released by Ultralytics in September 2024, is a real-time object detection model in the YOLO series [26]. Compared to YOLOv8 and YOLOv10, it features an optimized CSPDarknet backbone with refined channel allocation, a more efficient C2f neck enhanced for feature fusion, and a decoupled detection head that improves both classification and localization accuracy. Owing to its superior balance of speed and accuracy, the YOLO algorithm has been extensively applied in medical image analysis [27]. Many previous studies have optimized the YOLOv8 model for brain tumor detection tasks [18, 19, 28]. Compared to YOLOv8, the latest version of YOLOv11 exhibits excellent feature representation capability and detection performance on challenging visual tasks [26]. Therefore, in this study, YOLOv11 is employed as the baseline model for brain tumor detection in MRI scans.

For brain tumor detection in Magnetic Resonance Imaging (MRI), the original YOLOv11 network has difficulty effectively integrating discriminative feature information from MRI slices, resulting in an inability to accurately distinguish tumor lesions from normal brain tissues. Furthermore, the high visual similarity between tumor regions and background brain tissues in MRI images makes the model more susceptible to misleading information. Although YOLOv11 demonstrates superior performance compared with other mainstream algorithms in tumor detection accuracy, it still has clear limitations in detecting small-sized early-stage tumors and those with blurred boundaries. Therefore, further optimization of the algorithm design or targeted improvement strategies is required to boost lesion detection precision while preserving real-time inference performance. To overcome the limitations of the baseline YOLOv11 model in brain tumor detection, an improved framework, BrainYOLO-MCA, is developed by enhancing feature extraction, small-lesion perception, and feature fusion efficiency. The proposed framework incorporates an attention-enhanced backbone, a high-resolution detection branch, optimized dynamic upsampling, and sparse contextual modeling to improve lesion localization and representation. Together, these components improve detection robustness for challenging MRI tumor patterns while maintaining computational efficiency. The complete architecture of BrainYOLO-MCA is illustrated in Fig. (**2**), while each component is described in the following subsections.

#### 2.2.2 DySample Module

**Fig. (3):**
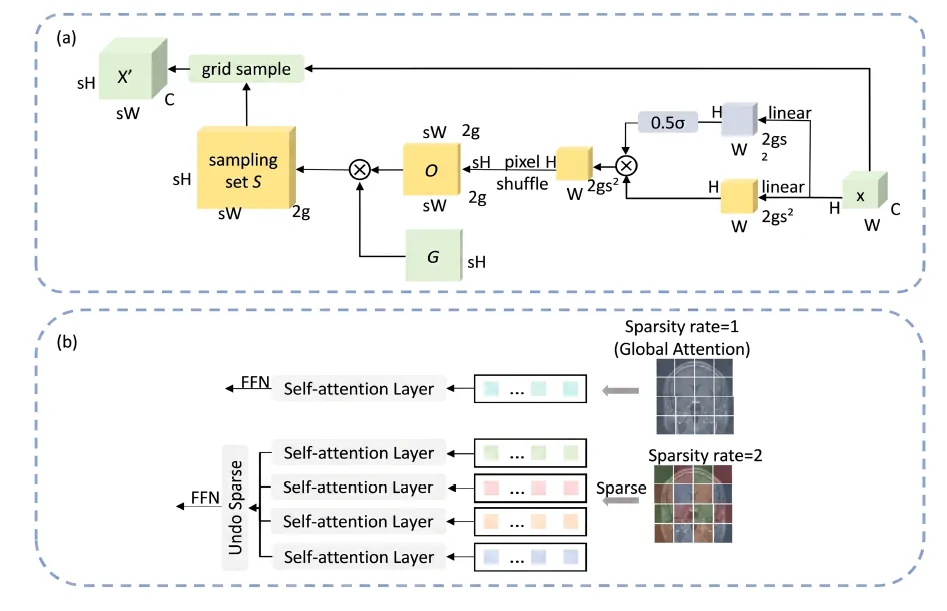
Schematic diagrams of the DySample module and the Sparse Self-Attention module. (**a**) DySample-based dynamic upsampling workflow and sampling point generation process based on the DySample method [29]. (**b**) Sparse Self-Attention mechanism, where self-attention is computed only among selected tensor blocks based on the sparse attention mechanism [22].

In brain MRI images, tiny early-stage tumors have very limited discriminative features, with edge contours being key diagnostic cues. However, conventional upsampling operations tend to blur these fine-grained details during feature scaling, causing irreversible feature loss in small lesions. To mitigate this problem, we adopt the point-based DySample upsampler [29] to improve the model's sensitivity to small intracranial tumors. The complete dynamic upsampling pipeline and sampling point generation process are shown in Figure **3a**. The sampling point generator takes the input feature map x and produces sampling points S. These points are resampled using a grid sampling module to yield the upsampled feature map x′. Figure **3b** shows the internal structure of the DySample sampling point generator. It implements two sampling mechanisms: static and dynamic range factor methods. The dynamic method uses a linear layer, pixel shuffling, and a learnable range factor. This factor, computed *via* a Sigmoid function, modulates the offset O. In the static method, the feature map is first processed through a linear layer with input and output channels of c and 2*s*^2^, respectively. The linear layer is then combined with pixel shuffling to generate the offset *O*, which is subsequently combined with the original sampling grid *G* to form the sampling set *S*. The core formulas of the DySample module are presented in Eqs. (**1**-**4**) [29]:


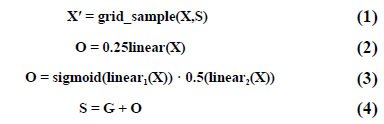


#### 2.2.3 Sparse Self - Attention module

To mitigate this issue, we integrate the sparse self-attention mechanism derived from the Swin Transformer architecture [22] into the feature extraction network. Sparse self-attention introduces the concept of “sparsity rate”(S) as a new structural hyperparameter. Given an input feature map *X*ϵR*^H×W×C^*, instead of computing attention across the entire *H*×*W* feature map, the features are divided into non-overlapping tensor blocks of size (*S*×*S*,*H*/*S*×*W*/*S*,C). Self-attention is computed independently within each of the non-overlapping tensor blocks of size *S*×*S* in the feature map divided into *H*/*S*×*W*/*S* regions. As illustrated in Fig. (**3b**), attention is performed only among tensor blocks of the same color, while blocks marked with different colors do not share attention computations. This mechanism reduces the model’s overreliance on semantic information, allowing it to focus more on extracting non-semantic, fine-grained features. Moreover, since attention is computed only within local tensor blocks, a significant amount of redundant computation is eliminated, reducing computational cost and improving overall efficiency [22].

#### 2.2.4 High-resolution Detection Head

In the original YOLOv11 network, the output feature map sizes of the backbone are 80 × 80, 40 × 40, and 20 × 20 [26]. Although continuous convolution and feature fusion enhance the model’s feature representation capability, they also reduce the spatial resolution of the feature map, resulting in each pixel in the deep feature map corresponding to a larger receptive field in the original image [30]. For small early-stage brain tumors, this leads to insufficient extraction of discriminative features and an increased likelihood of misclassification [31]. To address this issue, we optimize the network structure by adding an additional 160 × 160 detection layer. The main objective of this modification is to strengthen the model’s ability to detect small targets. Since the first C3k2 module in the backbone network is located in the lower layer and retains more fine-grained image details, we fuse the features from this C3k2 output with the features obtained through three successive upsampling operations at the 160 × 160 detection layer. This fusion allows for more precise detection of small brain tumors.

#### 2.2.5 Multi-scale Channel Attention module

In brain tumor detection, tumors with blurred boundaries often resemble surrounding brain tissues, leading to missed or incorrect detections, which significantly impacts the model’s accuracy. Most existing attention modules primarily emphasize semantic information, while neglecting non-semantic cues that are crucial for distinguishing tumors with unclear boundaries. To address this limitation, this study introduces the C3k2-MCA module, specifically designed to improve the detection of ambiguous and indistinct tumors. The module replaces one convolutional layer in the traditional BottleNeck structure with the proposed Multi-Scale Channel Attention (MCA) module, thereby forming an enhanced BottleNeck-MCA, which is then integrated into C3k2-MCA. During spatial information aggregation, average pooling is employed to compress the spatial dimensions of the input feature maps, enabling efficient computation of channel attention. Additionally, max pooling is used to capture distinctive object features, facilitating more precise inference of channel attention [32].

Feature extraction errors during convolution mainly originate from two sources: Increased estimation variance caused by the limited receptive field size. Mean bias introduced by convolutional parameter errors. Average pooling can mitigate the variance increase caused by limited receptive fields by preserving more background information, whereas max pooling reduces mean bias by retaining more texture details [32]. Therefore, by combining the results of max pooling and average pooling, the model can simultaneously minimize both sources of error. Subsequently, fusing the background information (from average pooling) and texture information (from max pooling) enables the extraction of more discriminative features, thereby achieving improved tumor boundary recognition and detection accuracy [32].

The proposed Multi-Scale Channel Attention (MCA) module enhances the original C3k2 structure by processing the input feature map z ϵ ℝ^𝐻×𝑊×𝐶^ through parallel max pooling and average pooling branches. These two pooling methods provide distinct spatial context descriptions of the feature map, effectively capturing different aspects of spatial information. Next, the two spatial context representations are separately processed through multi-scale convolutions. Specifically, each input feature map undergoes convolutions with 3×3 and 5×5 kernels to extract features at different receptive field sizes. The outputs of the 3×3 and 5×5 convolutions are then summed together with the original feature map to emphasize important features. These kernels, operating at multiple receptive field scales within the same network layer, allow the model to capture effective multi-scale features from both pooled representations, thereby enhancing the network’s feature representation capability. Afterward, the combined results are fused using a 1×1 convolution, and the two fused outputs (from max and average pooling branches) are added together and multiplied by the input feature map to complete the channel-wise information exchange. This design enables comprehensive fusion of multi-scale features derived from both pooling operations and allows the model to learn richer and more discriminative feature representations during training. The mathematical formulation of these operations is expressed in Eqs. (**5**-**8**):


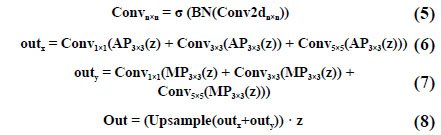


Here, MP denotes the Max Pooling operation, and AP denotes the Average Pooling operation. The symbol *σ* represents the SiLU activation function, which introduces non-linearity while maintaining smooth gradients for efficient optimization. BN stands for the BatchNorm2d layer, which normalizes feature distributions across mini-batches to accelerate convergence and improve model stability.

Experiments confirm that the C3k2-MCA-enhanced YOLO11 model effectively handles regions where tumors are easily confused with normal brain parenchyma. It achieves outstanding performance in the tests conducted on both the Br35H and MBrT datasets. A schematic diagram of the Multi-Scale Channel Attention (MCA) module is presented in Fig. (**4**).

### 2.3 Evaluation Metrics

Model performance was evaluated using five standard object detection metrics, including Precision (P), Recall (R), Intersection over Union (IoU), mAP@0.5, and mAP@0.5:0.95 [15, 30]. In this study, TP, TN, FP, and FN denote true positive, true negative, false positive, and false negative predictions, respectively. The corresponding evaluation metrics are defined as follows:

Precision (P) is defined as the ratio of true positive samples to the total number of samples classified as positive, as shown in Eq. (**9**):


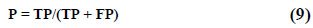


Recall (R) represents the proportion of actual positive samples that are correctly predicted as positive by the model, as shown in Eq. (**10**):


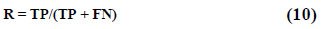


Intersection over Union (IoU) measures the degree of overlap between bounding boxes and quantifies how well the predicted box aligns with the ground truth box, as shown in Eq. (**11**):


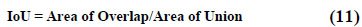


Mean Average Precision (mAP), including mAP@0.5 and mAP@0.5:0.95, was used for performance evaluation. The latter reflects average detection performance over IoU thresholds from 0.5 to 0.95 and is computed using Eq. (**12**):


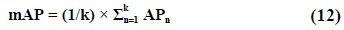


## 3 RESULTS AND DISCUSSION

### 3.1 Experimental Environment

The experimental environment for this study is as follows: Hardware: The proposed model was trained on an NVIDIA GeForce RTX 3080 GPU, with an Intel(R) Core(TM) Ultra7 265K @ 3.90 GHz CPU and 32 GB of RAM. Software: The operating system is Windows 10, using CUDA 12.1, Python 3.10, and PyTorch 2.0.0.Hyperparameters: The optimizer is SGD with a learning rate of 0.01 and momentum of 0.937. Input image size is 640 × 640, batch size is 16, and the model was trained for 200 epochs. During both the training and inference phases, all original brain MRI images were first resized to 640×640 pixels using bilinear interpolation before being fed into the network. No sliding window or tiling techniques were employed in this study.

### 3.2 Model Results and Discussion

#### 3.2.1 Results on the Br35H Dataset

**Fig. (5):**
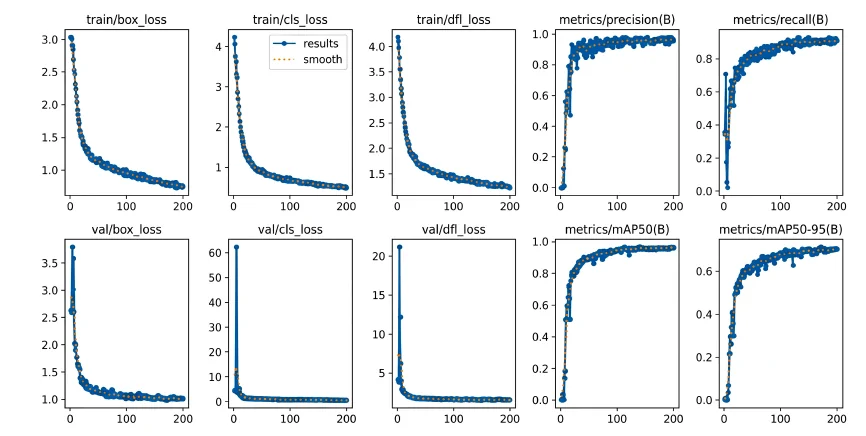
Results of the proposed model on the Br35H dataset.

Figure **5** shows stable convergence of the training and validation losses on the Br35H dataset, accompanied by progressive improvements in precision, recall, mAP50 and mAP50–95, indicating good optimization stability of the proposed model. The proposed method was further compared with YOLOv5 [33], YOLOv8 [28], YOLOv9 [34], YOLOv10 [35], YOLOv11 [26], BGF-YOLO [36], and RT-DETR_R50 [37]. As summarized in Table **2**, BrainYOLO-MCA achieves superior performance on the Br35H dataset across major evaluation metrics. All experimental results in this study represent the average of three independent training runs. Paired t-test analysis demonstrated that the performance improvements of the proposed model over the baseline model were statistically significant (*p* < 0.01).

From the detection and comparison results on the Br35H dataset, it can be observed that some models incorrectly detect normal tissue as tumor regions and produce redundant bounding boxes within the same area. In contrast, the model proposed in this study not only accurately identifies both small and large tumor lesions in brain MRI images but also achieves higher confidence scores. As shown in Fig. (**6**), the proposed model demonstrates outstanding performance on the Br35H dataset.

#### 3.2.2 Results on the MBrT Dataset

**Fig. (7):**
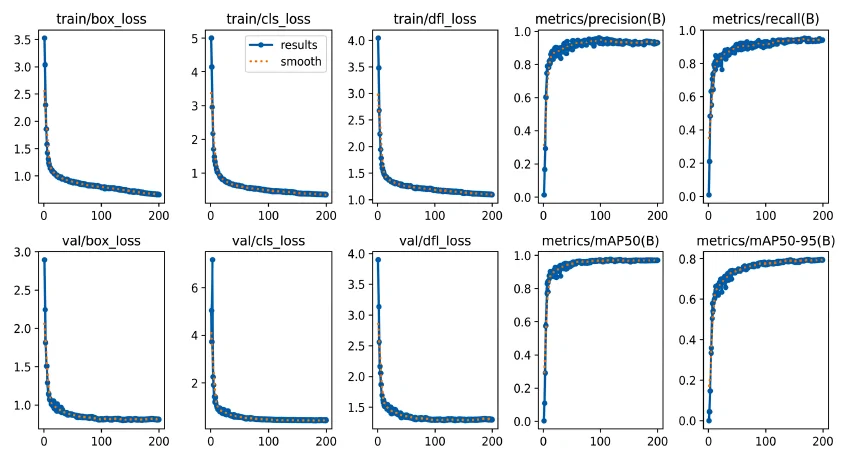
Results of the proposed model on the MBrT dataset.

Figure **7** shows stable convergence behavior and consistent improvement of evaluation metrics during training on the MBrT dataset. The proposed model was compared with YOLOv5 [33], YOLOv8 [28], YOLOv9 [34], YOLOv10 [35], YOLOv11 [26], BGF-YOLO [36], and RT-DETR_R50 [37], and the results in Table **3** demonstrate superior performance and strong robustness on this dataset.

From the detection and comparison results on the MBrT dataset, it is evident that the proposed model delivers superior performance. Compared with other models, the proposed approach not only achieves precise localization of brain tumors with diverse shapes but also produces higher confidence scores. These results further demonstrate that our model attains higher detection accuracy and stronger generalization capability than existing algorithms for brain tumor detection in MRI images, as shown in Fig. (**8**).

#### 3.2.3 Results on the Figshare Dataset

**Fig. (9):**
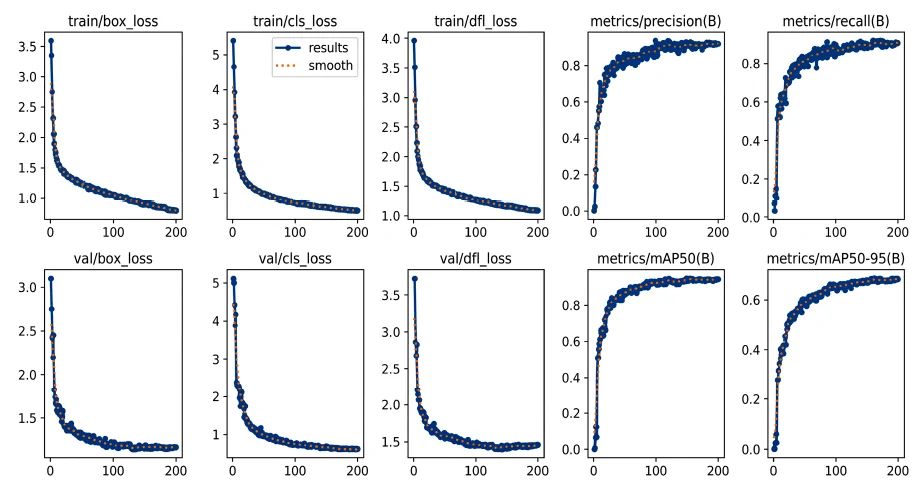
Results of the proposed model on the Figshare dataset.

Figure **9** shows favorable convergence characteristics of the proposed model on the Figshare dataset. The proposed model was compared with YOLOv5 [33], YOLOv8 [28], YOLOv9 [34], YOLOv10 [35], YOLOv11 [26], BGF-YOLO [36], RT-DETR_R50 [37], and ESA-YOLO [17]. Comparative results in Table **4** further verify that BrainYOLO-MCA achieves competitive and reliable detection performance on this dataset.

Comparative detection experiments conducted on the Figshare dataset further validate that the proposed model outperforms other methods, as presented in Fig. (**10**).

### 3.3 Comparison of Computational Costs with Other Brain Tumor Detection Models

Computational complexity and inference efficiency are critical factors for the clinical deployment of brain tumor detection models. Table **5** compares the performance of the proposed BrainYOLO-MCA model with state-of-the-art methods from four core dimensions: model storage requirement (number of parameters), theoretical computational complexity (GFLOPs), time required to process a single image (inference latency), and Frames Per Second (FPS). Compared with the baseline YOLOv11n, our model only increases by 0.6M parameters and 5.5 GFLOPs, yet achieves a maximum mAP50 improvement of 2.1% on the MBrT dataset. Meanwhile, it maintains a high inference speed of 146.2 FPS and a low inference latency of 6.87 ms, achieving an optimal balance between accuracy and efficiency and fully meeting the requirements of clinical deployment.

### 3.4 Ablation Study

To verify the effectiveness of the MCA module and other components in the proposed model, ablation studies were conducted on the Br35H, MBrT, and Figshare datasets using identical initialization parameters. In the experiments, “√” indicates that the corresponding module or dataset was incorporated into the YOLOv11 baseline model, whereas the absence of “√” denotes the use of the original YOLOv11 configuration. The ablation results are summarized in Table **6**. After integrating the proposed Multi-Scale Channel Attention module, the model achieves improvements over the original YOLOv11 network on the Br35H dataset, with increases of 0.4%, 0.5%, 1.6%, and 1.4% in precision (P), recall (R), mAP50, and mAP50–95, respectively. On the MBrT dataset, P, R, mAP50, and mAP50–95 improve by 0.9%, 2.2%, 1.0%, and 1.1%, respectively. On the Figshare dataset, the corresponding improvements reach 2.5%, 0.8%, 1.3%, and 1.6%. Finally, the complete BrainYOLO-MCA model achieves P, R, mAP50, and mAP50–95 values of 98.6%, 92.1%, 97.5%, and 72.3% on the Br35H dataset; 95.3%, 94.5%, 97.5%, and 80.5% on the MBrT dataset; and 96.1%, 91.9%, 96.3%, and 71.0% on the Figshare dataset, respectively.

In addition, to further validate the effectiveness of the proposed method for brain tumor detection, we visualized the detection results and corresponding heatmaps of YOLOv11, YOLOv11 with the MCA module, and the complete BrainYOLO-MCA model. As shown in Fig. (**11**), the YOLOv11 network exhibits missed detection in the second image and falsely identifies normal brain tissue as a tumor in the third image. After incorporating the proposed Multi-Scale Channel Attention module into the YOLOv11 backbone, the model is able to more accurately distinguish brain tumors that closely resemble normal tissue in the second image and effectively resolves the false positive observed in the third image. Moreover, for the final image containing a tumor with blurred boundaries, the detection confidence increases from 74% to 94%. The heatmap results further indicate that the introduction of the MCA module significantly enhances the model’s attention to tumor regions, enabling more precise coverage of the lesion areas. Overall, both the detection results and heatmaps demonstrate that the proposed MCA module achieves the intended improvements, allowing the model to better detect tumors with appearances similar to normal tissue as well as tumors with indistinct boundaries.

### 3.5 Results

BrainYOLO-MCA demonstrates superior overall performance for brain tumor detection across the Br35H, MBrT, and Figshare datasets. Meanwhile, the use of three datasets from different sources and diverse data augmentation strategies also alleviated dataset bias to a certain extent. The proposed MCA module effectively improves feature discrimination for tumors with blurred boundaries, while the integration of DySample, Sparse Self-Attention, and the high-resolution detection head further enhances detection capability. Comparative experiments and ablation results verify that these architectural optimizations jointly improve accuracy, robustness, and computational efficiency, indicating the effectiveness of the proposed framework for intelligent brain tumor detection.

Although the proposed model shows strong performance, several limitations remain. First, the public datasets used may not fully represent the diversity of MRI images in real-world clinical settings, including differences in imaging protocols and image quality. Second, the model may produce false positives or false negatives in images with severe motion artifacts. Third, when multiple lesions are closely adjacent, the model may merge them into a single detection box. Future work may focus on several directions: training the model on larger and more diverse MRI datasets to further improve accuracy and efficiency; adopting advanced image processing techniques to enhance robustness on low-quality data; collaborating with medical institutions to refine the model based on real clinical data and feedback; and exploring transfer learning strategies to apply pretrained models to other datasets or related medical imaging tasks, thereby improving generalization capability and practical applicability.

The clinical implementation pathway of the proposed model is as follows: First, conduct multi-center real-world validation to evaluate its generalization ability. Subsequently, perform regulatory submission in accordance with the requirements of the FDA Software as a Medical Device (SaMD) and CE certification. Leveraging the advantages of the model's lightweight design and high real-time performance, develop a plugin compatible with existing PACS systems to automatically output detection results within 1 second after the completion of MRI scanning, without changing the existing workflow of radiologists. Finally, establish a continuous monitoring mechanism to iteratively optimize the model based on clinical feedback.

## 4 STUDY LIMITATIONS

This study has several limitations. First, the datasets used may not fully represent the diversity of MRI images encountered in real clinical environments, including variations in imaging protocols and quality. Second, although the proposed model achieves strong performance on public datasets, its generalization ability to unseen clinical data requires further validation. Future work will focus on incorporating larger and more diverse datasets and improving robustness under real-world conditions.

## CONCLUSION

This study proposes BrainYOLO-MCA, an improved YOLOv11 framework for brain tumor detection in MRI images. By incorporating the designed Multi-Scale Channel Attention (MCA) module, which exploits both max pooling and average pooling to extract and fuse multi-scale spatial information from feature maps, combined with the Sparse Self-Attention mechanism, the proposed method effectively reduces false positives and missed detections when identifying tumors with blurred boundaries or appearances similar to normal brain tissue. Experimental results demonstrate that the proposed model can accurately detect and localize various types of brain tumors in MRI images while exhibiting excellent overall detection performance. Compared with mainstream object detection models including YOLOv5, YOLOv8, YOLOv9, YOLOv10, the baseline YOLOv11, BGF-YOLO, and RT-DETR_R50, the proposed BrainYOLO-MCA approach achieves leading detection accuracy across all three public datasets, highlighting its strong potential for clinical computer-aided diagnosis applications.

## Figures and Tables

**Fig. (1):**
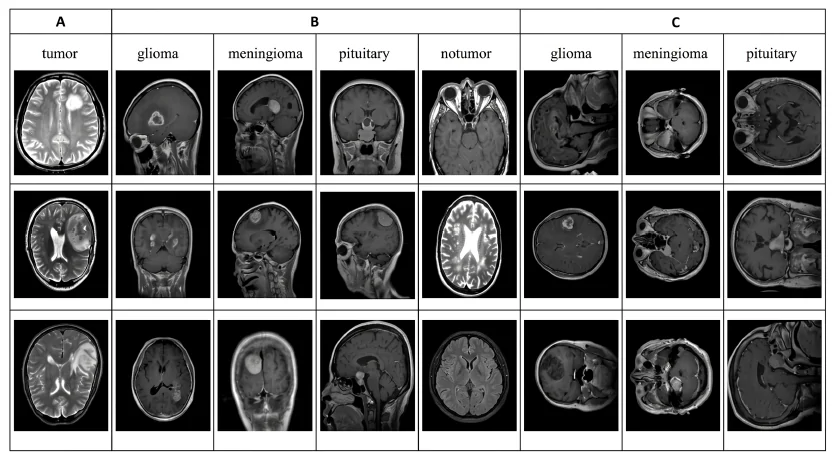
(**A**) shows sample images from the Br35H dataset. (**B**) presents images of the four categories glioma, meningioma, pituitary tumor, and no tumor from the MBrT dataset. (**C**) displays images of the three categories glioma, meningioma, and pituitary tumor from the Figshare dataset.

**Fig. (2):**
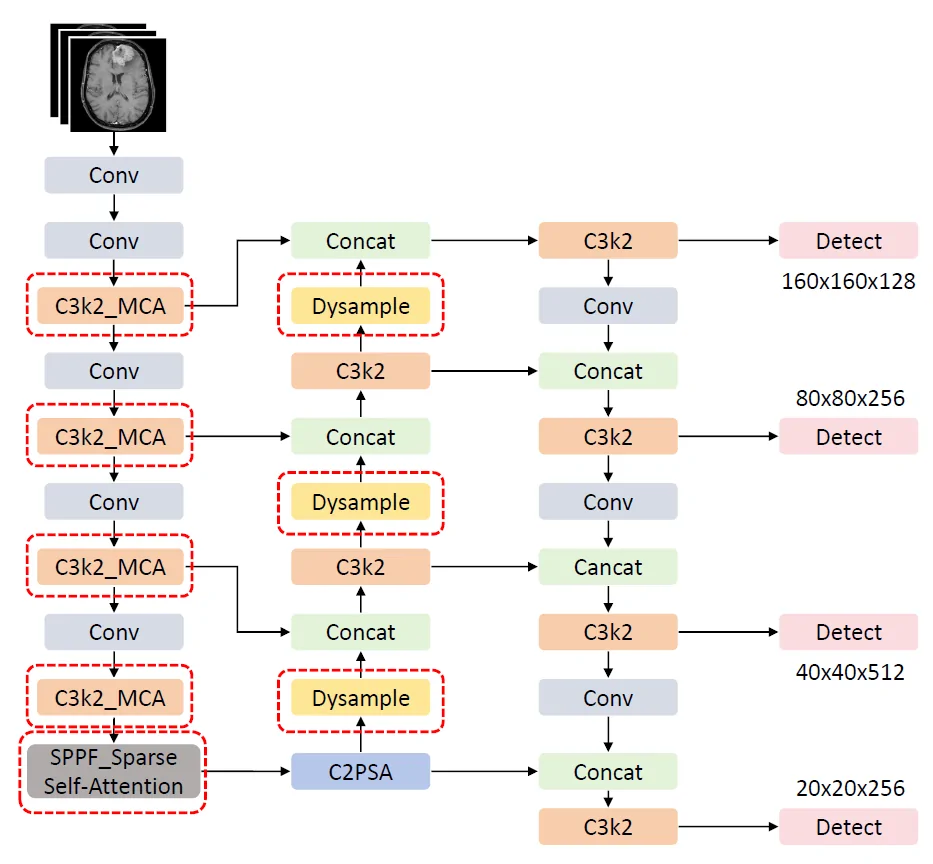
Architecture of the improved network, with the enhanced modules highlighted in red.

**Fig. (4):**
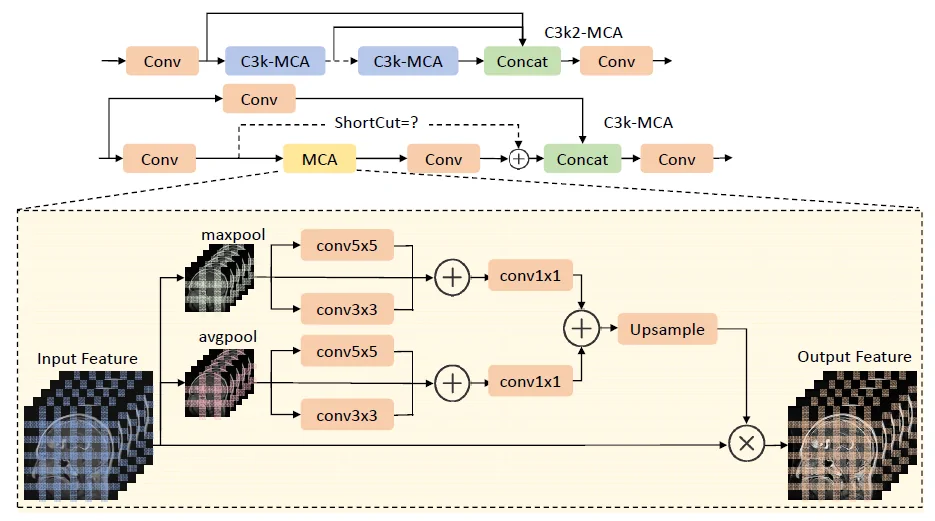
Illustration of the structure of the Multi-Scale Channel Attention (MCA) module.

**Fig. (6):**
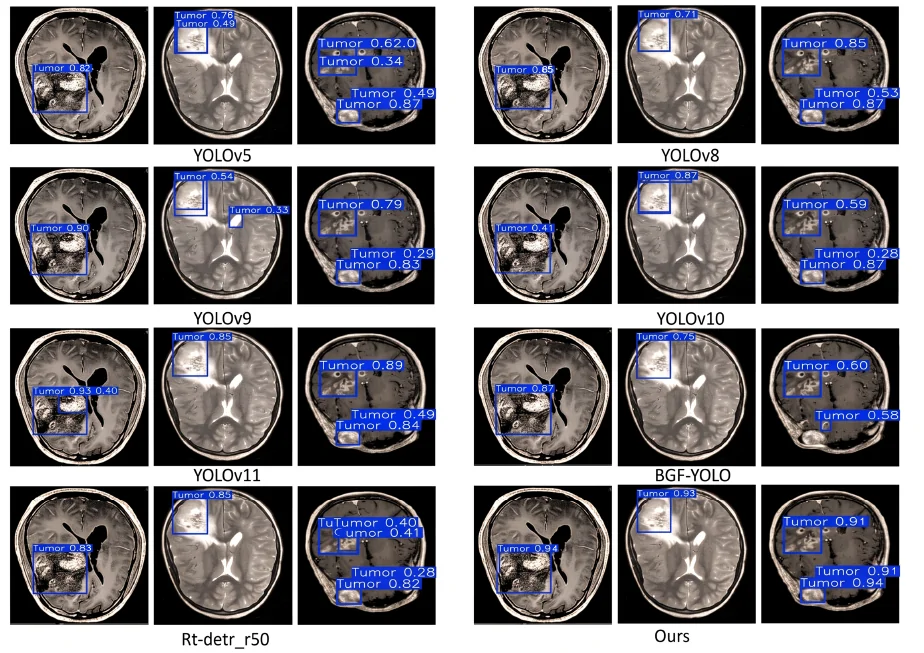
Example predictions of eight models on images from the Br35H dataset.

**Fig. (8):**
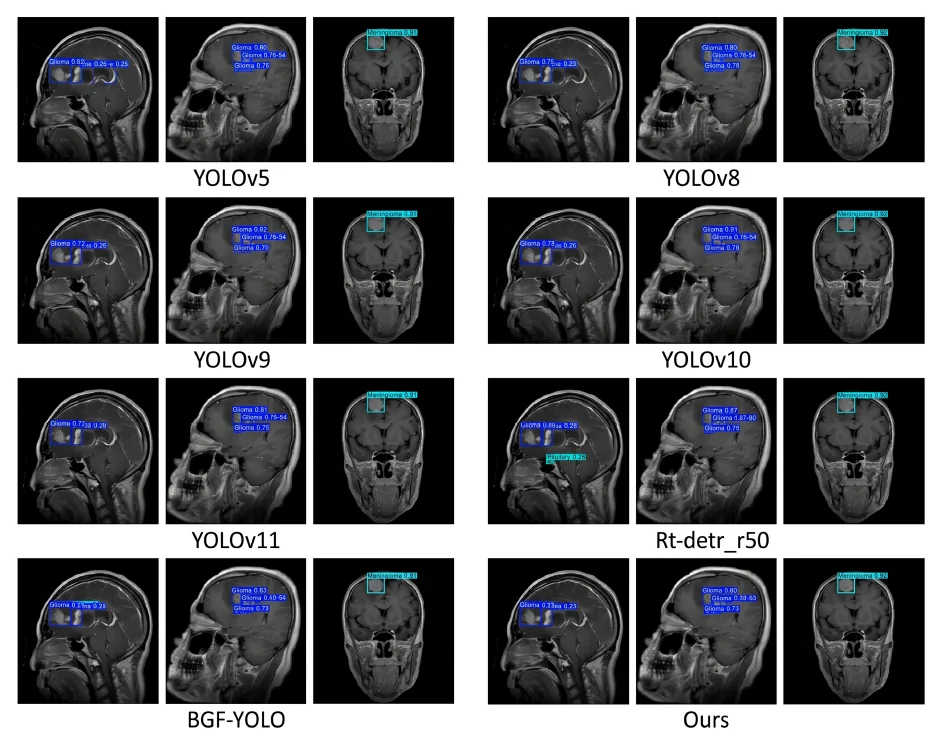
Example predictions of eight models on images from the MBrT dataset.

**Fig. (10):**
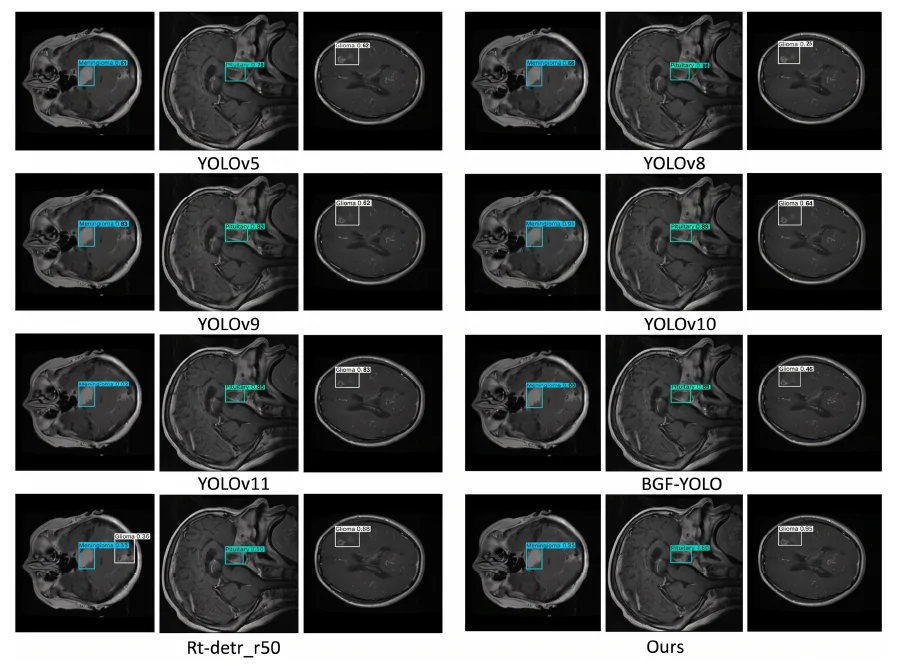
Example predictions of eight models on images from the Figshare dataset.

**Fig. (11):**
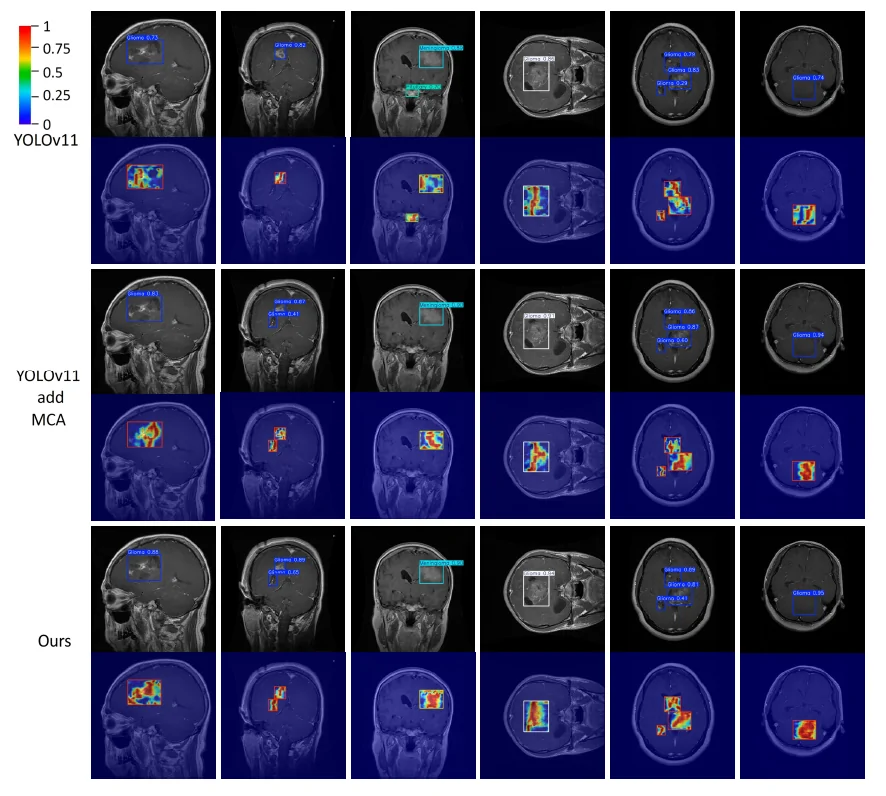
Detection results and heatmaps of YOLOv11, YOLOv11 with the MCA module, and our model.

**Table 1:** The dataset involved in the study.

**Data Set**	**Training Set**				**Testing Set**				**Validation Set**			
Br35H	640				81				80			
Category	Glioma	Meningioma	Pituitary tumor.	No tumor	Glioma	Meningioma	Pituitary tumor.	No tumor	Glioma	Meningioma	Pituitary tumor.	No tumor
MBrT	1015	1281	1261	642	121	156	166	83	135	138	166	86
Figshare	1137	560	753	-	144	72	91	-	145	76	86	-

**Table 2:** The results of the models based on the Br35H dataset.

**Models/Refs.**	**P(Precision)**	**R(Recall)**	**Map-50**	**Map50-95**
YOLOv5n [33]	0.962	0.875	0.939	0.689
YOLOv8n [28]	0.953	0.883	0.938	0.696
YOLOv9t [34]	0.941	0.861	0.935	0.685
YOLOv10n [35]	0.861	0.825	0.9	0.662
YOLOv11n [26]	0.979	0.912	0.954	0.701
BGF-YOLO [36]	0.99	0.832	0.953	0.687
Rt-detr_r50 [37]	0.871	0.779	0.897	0.662
Current Study	0.986	0.921	0.975	0.723

**Table 3:** The results of the models based on the MBrT dataset.

**Models/Refs.**	**P(Precision)**	**R(Recall)**	**Map-50**	**Map50-95**
YOLOv5n [33]	0.927	0.91	0.943	0.776
YOLOv8n [28]	0.937	0.929	0.957	0.781
YOLOv9t [34]	0.945	0.913	0.962	0.78
YOLOv10n [35]	0.927	0.894	0.953	0.764
YOLOv11n [26]	0.936	0.921	0.959	0.785
BGF-YOLO [36]	0.934	0.899	0.96	0.764
Rt-detr_r50 [37]	0.956	0.92	0.965	0.768
Current Study	0.953	0.945	0.975	0.805

**Table 4:** The results of the models based on the Figshare dataset.

**Models/Refs.**	**P(Precision)**	**R(Recall)**	**Map-50**	**Map50-95**
YOLOv5n [33]	0.901	0.861	0.923	0.653
YOLOv8n [28]	0.927	0.886	0.945	0.676
YOLOv9t [34]	0.91	0.905	0.935	0.679
YOLOv10n [35]	0.949	0.817	0.918	0.649
YOLOv11n [26]	0.923	0.904	0.942	0.682
BGF-YOLO [36]	0.906	0.897	0.93	0.672
Rt-detr_r50 [37]	0.946	0.88	0.93	0.668
ESA-YOLO [17]	0.92	0.878	0.924	-
Current Study	0.961	0.919	0.963	0.71

**Table 5:** Comparison of computational complexity and inference time between the proposed model and other brain tumor detection models. “GFLOPs” in the table refers to giga floating point operations, which measures the theoretical computational complexity of the model.

**Model**	**Params(M)**	**GFLOPs**	**Inference Latency(ms)**	**FPS**
YOLOv5n	2.5	7.2	3.46	292.1
YOLOv8n	3	8.2	3.2	312.6
YOLOv9t	2	7.8	9.95	102
YOLOv10n	2.7	8.4	5.1	197
YOLOv11n	2.5	6.4	4.32	233.5
BGF-YOLO	84.3	385.3	21.54	46.4
Rt-detr_r50	41.9	125.6	18.05	55.4
ESA-YOLO	22.1	106.3	19.3	51.8
Current Study	3.1	11.9	6.87	146.2

**Table 6:** The results of the models for the ablation study.

Br35H	MBrT	Figshare	C3k2 -MCA	AddDetect	DySample	Sparse Self Attention	Precision	Recall	Map-50	Map50-95
√	-	-	-	-	-	-	0.979	0.912	0.954	0.701
√	-	-	√	-	-	-	0.983	0.917	0.970	0.715
√	-	-	√	√	-	-	0.982	0.915	0.969	0.716
√	-	-	√	√	√	-	0.979	0.919	0.972	0.715
√	-	-	√	√	√	√	0.986	0.921	0.975	0.723
	√	-	-	-	-	-	0.936	0.921	0.959	0.785
	√	-	√	-	-	-	0.945	0.943	0.969	0.796
	√	-	√	√	-	-	0.949	0.942	0.968	0.798
	√	-	√	√	√	-	0.951	0.940	0.972	0.796
	√	-	√	√	√	√	0.953	0.945	0.975	0.805
	-	√	-	-	-	-	0.923	0.904	0.942	0.682
	-	√	√	-	-	-	0.948	0.912	0.955	0.698
	-	√	√	√	-	-	0.953	0.911	0.961	0.695
	-	√	√	√	√	-	0.955	0.92	0.96	0.704
	-	√	√	√	√	√	0.961	0.919	0.963	0.71

## Data Availability

The datasets used in this study are publicly available. The Br35H dataset is available at https://universe.roboflow.com/hashira-fhxpj/br35h-::-brain-tumor-detection-2020. The MBrT dataset is available at https://www.kaggle.com/datasets/ahmedsorour1/mri-for-brain-tumor-with-bounding-boxes. The Figshare brain tumor dataset is available at https://www.kaggle.com/datasets/ashkhagan/figshare-brain-tumor-dataset. These datasets are accessible for research purposes under their respective licenses. The supporting information includes the complete source code of the proposed BrainYOLO-MCA model. All resources are publicly available at https://github.com/withlight07/BrainYOLO-MCA.

## LIST OF ABBREVIATIONS

AI: Artificial Intelligence
AP: Average Pooling
BGF-YOLO: YOLO with Bidirectional Gradient Flow
BN: Batch Normalization
CBAM: Convolutional Block Attention Module
CNN: Convolutional Neural Network
CPU: Central Processing Unit
CUDA: Compute Unified Device Architecture
DySample: Dynamic Sampling-based Upsampling
ESA: Enhanced Spatial Attention
FDA: Food and Drug Administration
FN: False Negative
FP: False Positive
FPS: Frames Per Second
GFLOPs: Giga Floating Point Operations
GPU: Graphics Processing Unit
IoU: Intersection over Union
mAP: Mean Average Precision
MCA: Multi-Scale Channel Attention
MBrT: MRI for Brain Tumor with Bounding Boxes
MRI: Magnetic Resonance Imaging
PACS: Picture Archiving and Communication System
RAM: Random Access Memory
R-CNN: Region-based Convolutional Neural Network
RCS-YOLO: Re-parameterized Context-Sharing YOLO
RT-DETR: Real-Time Detection Transformer
SaMD: Software as a Medical Device
SGD: Stochastic Gradient Descent
SSD: Single Shot MultiBox Detector
SVM: Support Vector Machine
TN: True Negative
TP: True Positive
YOLO: You Only Look Once

## STANDARDS OF REPORTING

TRIPOD+AI guidelines were followed.
